# Development and Validation of a Nomogram to Predict the 180-Day Readmission Risk for Chronic Heart Failure: A Multicenter Prospective Study

**DOI:** 10.3389/fcvm.2021.731730

**Published:** 2021-09-07

**Authors:** Shanshan Gao, Gang Yin, Qing Xia, Guihai Wu, Jinxiu Zhu, Nan Lu, Jingyi Yan, Xuerui Tan

**Affiliations:** ^1^Clinical Research Center, The First Affiliated Hospital of Shantou University Medical College (SUMC), Cardiology, Shantou, China; ^2^Heart Failure center, Qingdao Central Hospital, Cardiology, Qingdao, China

**Keywords:** chronic heart failure, readmission, hospitalization, nomogram, prediction model

## Abstract

**Background:** The existing prediction models lack the generalized applicability for chronic heart failure (CHF) readmission. We aimed to develop and validate a widely applicable nomogram for the prediction of 180-day readmission to the patients.

**Methods:** We prospectively enrolled 2,980 consecutive patients with CHF from two hospitals. A nomogram was created to predict 180-day readmission based on the selected variables. The patients were divided into three datasets for development, internal validation, and external validation (mean age: 74.2 ± 14.1, 73.8 ± 14.2, and 71.0 ± 11.7 years, respectively; sex: 50.2, 48.8, and 55.2% male, respectively). At baseline, 102 variables were submitted to the least absolute shrinkage and selection operator (Lasso) regression algorithm for variable selection. The selected variables were processed by the multivariable Cox proportional hazards regression modeling combined with univariate analysis and stepwise regression. The model was evaluated by the concordance index (C-index) and calibration plot. Finally, the nomogram was provided to visualize the results. The improvement in the regression model was calculated by the net reclassification index (NRI) (with tenfold cross-validation and 200 bootstraps).

**Results:** Among the selected 2,980 patients, 1,696 (56.9%) were readmitted within 180 days, and 1,502 (50.4%) were men. A nomogram was established by the results of Lasso regression, univariate analysis, stepwise regression and multivariate Cox regression, as well as variables with clinical significance. The values of the C-index were 0.75 [95% confidence interval (CI): 0.72–0.79], 0.75 [95% CI: 0.69–0.81], and 0.73 [95% CI: 0.64–0.83] for the development, internal validation, and external validation datasets, respectively. Calibration plots were provided for both the internal and external validation sets. Five variables including history of acute heart failure, emergency department visit, age, blood urea nitrogen level, and beta blocker usage were considered in the final prediction model. When adding variables involving hospital discharge way, alcohol taken and left bundle branch block, the calculated values of NRI demonstrated no significant improvements.

**Conclusions:** A nomogram for the prediction of 180-day readmission of patients with CHF was developed and validated based on five variables. The proposed methodology can improve the accurate prediction of patient readmission and have the wide applications for CHF.

## Introduction

It is well-known that chronic heart failure (CHF) is a systemic clinical syndrome as well as an endpoint stage of various cardiovascular diseases with typical symptoms of systolic and/or diastolic dysfunction. In recent years, existing treatments are effective in reversing the progression to end-stage disease among patients diagnosed with CHF; however, the mortality and readmission rates are still high (1). To better address these issues, many experts and clinical physicians have committed to identifying a prognostic model of CHF in its early stages (2).

In developed countries, some physicians in the United States focus on the 30-day readmission rate (3, 4), thus leading to the investigation on the readmission rate in patients with CHF (5), which usually reflects the 30-day all-cause readmission or cardiovascular outcomes (6). However, the focus varies in developing countries (7). The existing readmission models of heart failure (HF) have been established and widely used in different regions of China, including the standard model developed by Tan et al. (8) and two models restricted to the northern cities of China (9, 10). Given the delay in the treatment of patients, the 180-day readmission time seems more suitable for patients in developing countries (11, 12). Based on these studies, we can conclude that readmission rates vary from 34.9 to 56.4%, and most of the state-of-the-art models are derived from single-center studies consisting of a large sample of patients admitted in the hospital, which usually lack general applicability in real-world clinical diagnoses. The objective of this study is to establish a widely applicable 180-day readmission nomogram for patients with CHF in North and South China.

## Materials and Methods

**Figure 1:**
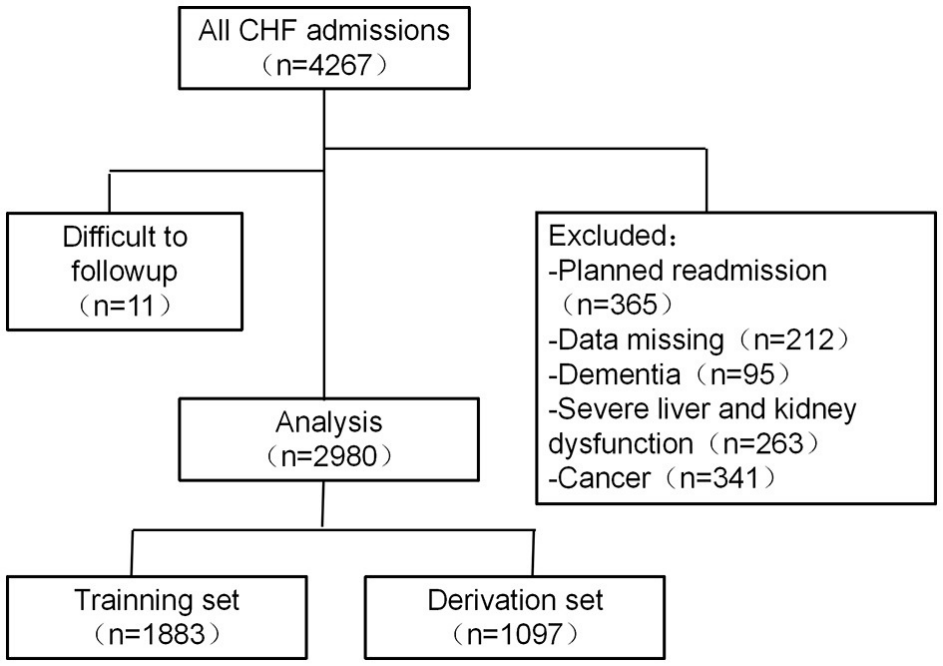
Flowchart of the chronic heart failure prediction model.

The flowchart of the selection of patients for the multicenter prospective cohort study is depicted in Figure 1. First, the collected samples from a medical center of North China were divided into development and internal validation databases, and then a nomogram was proposed for the research works. Second, the models were evaluated by using an interval validation set. Third, the data provided by a medical center in South China were used for external validation. Following the steps mentioned above, it is natural for us to develop effective methods.

### Study Population

We selected patients hospitalized for CHF between January 2019 and June 2019 at two centers, where one was a North China center of HF (Qingdao Central Hospital) and the other was a South China center of HF (The First Affiliated Hospital of Shantou University Medical College). The patients would be enrolled if they met the following criteria: patients with CHF and diagnosed with various etiologies, New York Heart Association (NYHA) class II–IV, and age >18 years. Moreover, the exclusion criteria were as follows: planned HF readmission within 6 months (patients who had hospitalization plan within 6 months were excluded), missing data for the essential variables and outcomes (patients missing 30% or more variables will be excluded), severe patients with liver and kidney dysfunction and/or malignant tumors, and poorer treatment compliance (i.e., difficult to follow up). Severe liver dysfunction is defined as an alanine aminotransferase level more than twice that of normal or Budd–Chiari grade C. Severe renal dysfunction is defined as chronic kidney disease stage V. As per the above indicators, the definitions are provided in further studies.

It should be stressed that this study was approved by the ethics committee (KY-2018046, No. 2019129) of each chosen hospital and was also conducted in accordance with the Declaration of Helsinki (ChiCTR1800019869). Furthermore, the participants from both hospitals provided informed written consent for supporting the clinical research studies.

### Predictors

In this study, the main predictors include sociodemographics, clinical characteristics, comorbidities, results of laboratory tests (blood sample assay), other relevant physical examinations, medication usages in the hospital and at discharge, and other treatment measures. In detail, the sociodemographics contain basic information, such as age, sex, the distance between the hospital and residence, length of hospital stay, medical social insurance status, and other variables associated with readmission. In addition, we collected some samples in the progress of treatments, as detailed as possible, and selected the features that corresponded to the prognosis of the disease. Among these predictors, comorbidities, complications, and Charlson comorbidity index (CCI) (13) were recorded for each patient.

### Definitions

The diagnosis of CHF is based on the 2016 ESC Guideline for HF management, and patients were diagnosed by two experienced cardiologists in each HF center (1). The 180-day cardiovascular readmission is defined as 180-day unplanned readmission, registered in the same HF center as the index hospitalization (when HF worsens, the patients will be transferred from the community hospital to the nearest HF center). It should be mentioned that estimated glomerular filtration rate (eGFR), as a baseline indicator, reflects the filtration function of the kidney and mortality due to heart failure with preserved ejection fraction (HFpEF) (14). In the numerical results, eGFR is calculated by the Chronic Kidney Disease Epidemiology Collaboration formulas shown below:

$$\begin{array}{l} \text{eGFR = 141}\times \text{min (}Cr/k\text{,1}{\text{)}}^{\alpha }\;\times \text{max (}Cr/k\text{,1}{\text{)}}^{\text{-1.209}}\;\times \text{0.99}{\text{3}}^{\text{Age}}\;\times \\ \;\text{1.018}\left(\text{Female}\right)\times \text{1.159 (Black),} \end{array}$$

where*k* = 0.7 (Female) or 0.9 (Male), α = -0.329(Female) or-0.411 (Male), *Cr* = plasma creatinine

$$\begin{array}{l} \left(\text{mg/dl}\right) \end{array}$$

1 mg/dl = 88.4 μmol/l (15).

### Model Development Cohorts

The prediction model for CHF can be established according to the statements in the transparent reporting of a multivariable prediction model for individual prognosis (TRIPOD) guidelines (16), where we use the data samples from a prospective cohort study at the HF center of Qingdao Central Hospital. To satisfy the study requirements, we identified 2,687 consecutive patients with HF and then randomly divided them into development and validation cohorts by virtue of a ratio of 7:3. The development cohort (comprising 1,883 patients, i.e., 70% of the Qingdao Central Hospital cohort) was used for the model development.

### Model Validation Cohorts

In addition to the internal validation cohorts (comprising 804 patients, i.e., 30% of the Qingdao Central Hospital cohort), an external validation cohort (comprising 293 patients) was developed from a prospective study at the First Affiliated Hospital of Medical College in Shantou University. Based on the development and validation cohorts, numerical comparisons will be provided by the proposed methodology.

### Outcome

The primary outcome is unplanned HF readmission within 180 days after the first admission for the referred patients. They are collected from both centers mentioned above, and they are followed up for 180 days after discharge from the index admissions. To better track the patients, we adopted the way by telephoning the interviews and further verified their conditions by virtue of the hospital system.

### Statistical Analysis

**Figure 2:**
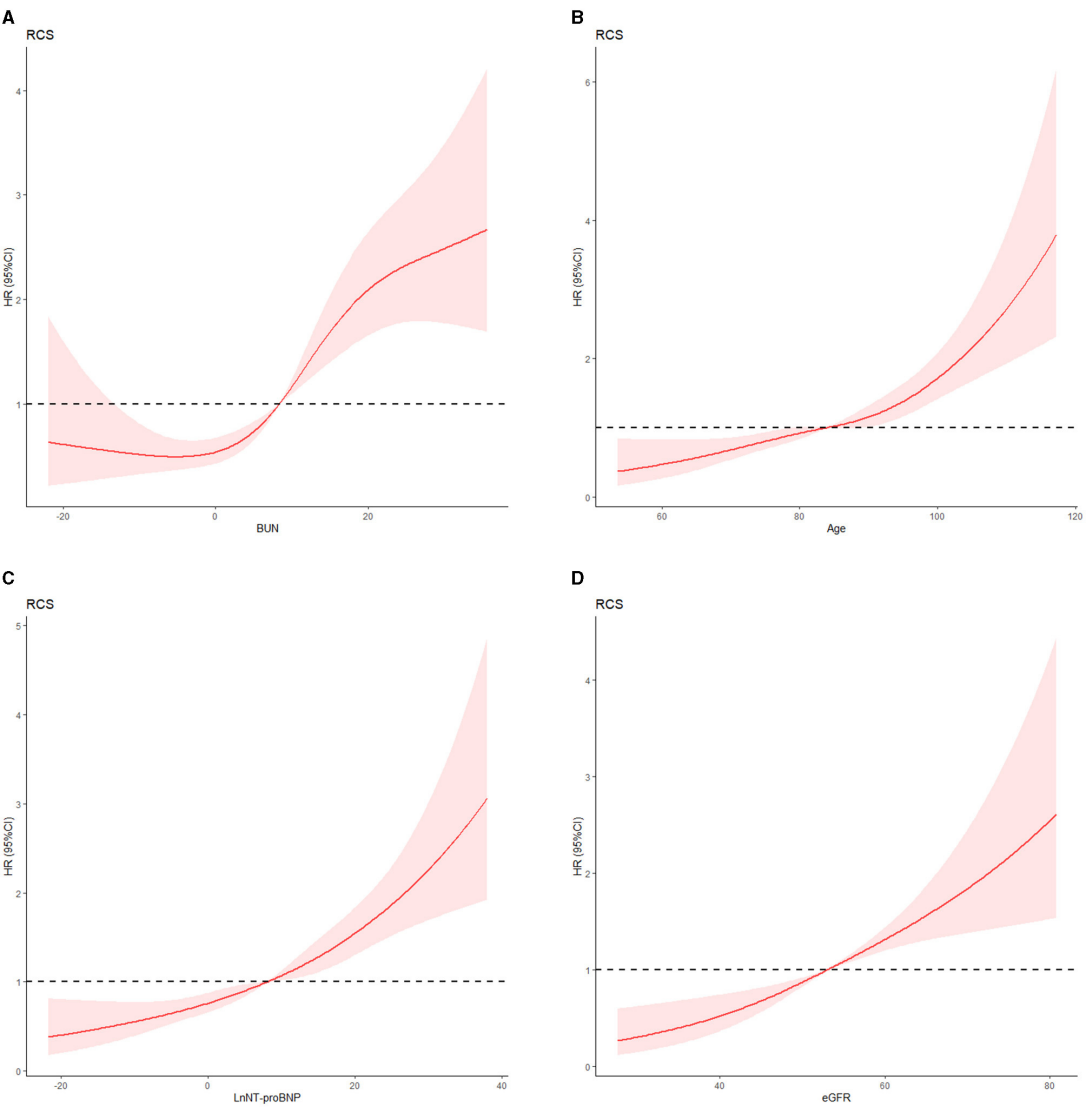
**(A)** Blood urea nitrogen level and 180-day heart failure readmission risk. **(B)** Age and 180-day heart failure readmission risk. **(C)** Natural logarithm of plasma N-terminal pro-B-type natriuretic peptide (LnNT-proBNP) and 180-day heart failure readmission risk. **(D)** Estimated glomerular filtration rate and 180-day heart failure readmission risk. We used a three-level classification of BUN, eGFR, and LnNT-proBNP and five-level classification of age according to the results of restricted cubic spline and clinical meanings.

We provide the numerical comparisons for the continuous variables, which are described as the mean ± standard deviation. Meanwhile, categorical variables are described as percentages or frequencies. Continuous variables are divided into categorical variables based on the results of restricted cubic spline according to data distribution and its clinical implications and boundaries (Figures 2A–D). Hazard ratios (HRs) are expressed as 95% confidence interval (CI). The least absolute shrinkage and selection operator (Lasso) regression-based mathematical model would be considered to screen the variables, and the multivariate Cox regression analysis is presented to generate a prediction model for patients with CHF. Meanwhile, taking the results of univariate Cox and stepwise regression into consideration, they are combined with clinical significance. The improvement of the model is evaluated by net reclassification improvement (NRI) (17) when adding variables to the model. The involved variables with missing values are <30% of the development population, and they are selected to enter into the Lasso and Cox regression models for further analysis. To make comparisons, 10-fold cross-validation operations are presented in the model development set. Finally, the missing values are imputed through multiple imputation in the statistical analysis.

In the numerical verification, both internal and external comparisons are conducted for the desired expectations. The concordance index (C-index), with 200 bootstrap samples, and the calibration plot are considered to evaluate the prediction accuracy as well as test the consistency of the prediction model, respectively. Besides this, the NRI is provided to compare the different models in the numerical experiments. All of the statistical analyses are conducted using SPSS version 25.0 (IBM Corp., Armonk, NY) and the R Project package for statistical computing (www.cran.r-project.org/version 3.6.1) on a personal computer.

## Results

### Baseline Characteristics

A total of 2,980 patients were enrolled between January 2019 and June 2019. The baseline characteristics of the patients are shown in Table 1. In the entire cohort, 1,696 (56.9%) patients were readmitted within 180 days. The 180-day readmission rates were 59.0% (1,112 patients), 54.1% (435 patients), and 51.2% (150 patients) in the development, internal validation, and external validation cohorts, respectively. The mean ages were 74.2 ± 14.1 years in the development cohort, 73.8 ± 14.2 years in the internal validation cohort, and 71.0 ± 11.7 years in the external validation cohort. Male patients accounted for 50.4% (1,502) of the patients in the entire cohort; 1,345 (45.1%) had coronary heart disease and 1,795 (60.2%) had a history of acute heart failure (AHF). The proportion of HFrEF is 31.3%, both in the development set and internal validation set. In the external validation set, the rate is 12.9%. Based on previously published data, patients with HFpEF and patients with HFrEF often have similar predictors (18). Consequently, they are all included in our study. We made a collinearity diagnosis of prediction variables at baseline and excluded the variables with no clinical significance and correlation coefficient >0.7.

**Table 1:** Baseline characteristics of the enrolled patients with CHF.

**Characteristic**	**Development set**	**Internal vad**	**External vad**	***P-*Value**
	**(*n* = 1,883)**	**(*n* = 804)**	**(*n* = 293)**	
Age, years (mean ± SD)	74.2 ± 14.1	73.8 ± 14.2	71.0 ± 11.7	0.089
Gender: Male, *n* (%)	947 (50.2%)	393 (48.8%)	162 (55.2%)	0.169
HFrEF, *n* (%)	590 (31.3%)	252 (31.3%)	38 (12.9%)	<0.01
SBP (mmHg), mean ± SD	148 ± 17	132 ± 7	147 ± 33	<0.01
DBP (mmHg), mean ± SD	81 ± 8	76 ± 2	89 ± 13	<0.01
Remoteness (<5 km), *n* (%)	1,653 (87.8%)	713 (88.6%)	142 (48.4%)	<0.01
Hospital stay (day), mean ± SD	15 ± 5	12 ± 2	5 ± 2	<0.01
CHD, *n* (%)	898 (47.6%)	376 (46.7%)	71 (24.2%)	<0.01
AHF, *n* (%)	1,204 (63.9%)	484 (60.1%)	107 (36.5%)	<0.01
Hypertension, *n* (%)	1,547 (82.1%)	658 (81.8%)	256 (87.3%)	0.07
Diabetes, *n* (%)	716 (38.0%)	310 (38.5%)	115 (39.2%)	<0.01
Alcohol consumption, *n* (%)	354 (18.7%)	150 (18.6%)	18 (6.1%)	<0.01
Smoker, *n* (%)	363 (19.2%)	157 (19.5%)	64 (21.8%)	0.433
COPD, *n* (%)	179 (9.5%)	60 (7.4%)	32 (10.9%)	0.146
Pulmonary infection (not COVID-19), *n* (%)	220 (11.6%)	81 (10%)	67 (22.8%)	<0.01
AF, *n* (%)	709 (37.6%)	275 (34.2%)	73 (24.9%)	<0.01
CCI, mean ± SD	4.0 ± 0.5	4.5 ± 1.5	4.5 ± 0.5	<0.01
Stroke, *n* (%)	200 (10.6%)	118 (14.6%)	47 (16.0%)	0.076
Hypertensive heart disease, *n* (%)	805 (42.7%)	318 (39.5%)	85 (29.0%)	<0.01
Hyperthyroidism, *n* (%)	56 (2.9%)	23 (2.8%)	6 (2.0%)	0.07
Hyperkalemia, *n* (%)	105 (5.5%)	35 (4.3%)	9 (3.0%)	0.292
WBC (*10∧9/L), mean ± SD	6.0 ± 1.02	6.7 ± 0.5	4.2 ± 2.73	0.012
RBC (*10∧12/L), mean ± SD	4.0 ± 0.5	5.2 ± 0.7	4.1 ± 0.2	<0.01
Hemoglobin (g/L), mean ± SD	144 ± 4	127 ± 6	119 ± 19	<0.01
RDW-CV (%)	13.6 ± 1.1	9.9 ± 1.0	16.7 ± 0.8	<0.01
Glucose (mmol/L), mean ± SD	9.6 ± 0.5	4.7 ± 0.1	8.7 ± 3.6	<0.01
Serum sodium (mmol/L), mean ± SD	140 ± 4	142 ± 3	135 ± 7.9	<0.01
Serum potassium (mmol/L), mean ± SD	3.9 ± 0.8	4.3 ± 0.1	4.1 ± 0.1	<0.01
TC (mmol/L), mean ± SD	4.52 ± 1.51	3.75 ± 0.54	1.5 ± 0.3	<0.01
NTproBNP (pg/mL), mean ± SD	8,720 ± 4,914	9,669 ± 9,479	500	<0.01
eGFR (mL/min/1.73 m^2^), mean ± SD	34 ± 9	57 ± 31	46 ± 3	<0.01
Creatinine (μmol/L), mean ± SD	82 ± 3	203 ± 74	109 ± 4	<0.01
BUN (mmol/L), mean ± SD	12.9 ± 5.5	8.0 ± 0.8	7.2 ± 0.6	<0.01
CysC (mg/L), mean ± SD	0.8 ± 0.1	1.1 ± 0.3	1.31	0.037
UA (μmol/L), mean ± SD	330 ± 117	323 ± 167	382 ± 66	<0.01
Albumin (g/L), mean ± SD	44 ± 7	37 ± 9	32.8 ± 6.9	<0.01
ACEI, *n* (%)	336 (17.8%)	157 (19.5%)	76 (25.9%)	<0.01
ARB, *n* (%)	962 (51.0%)	403 (50.1%)	106 (36.1%)	<0.01
Beta-blocker, *n* (%)	1,216 (64.5%)	531 (66%)	134 (45.7%)	<0.01
Diuretic, *n* (%)	1,362 (72.3%)	572 (71.1%)	148 (50.5%)	<0.01
MRA, *n* (%)	1,605 (85.2)	683 (84.9%)	127 (43.3%)	<0.01
ARNI, *n* (%)	585 (31%)	244 (30.3%)	12 (4.0%)	<0.01
Anti-PLT medication, *n* (%)	1,193 (63.3%)	537 (66.7%)	77 (26.2%)	<0.01
CCB, *n* (%)	587 (31.1%)	257 (31.9%)	169 (57.6%)	<0.01
Antidiabetic drugs, *n* (%)	578 (30.6%)	243 (30.2%)	88 (30.0%)	0.955
Statin, *n* (%)	602 (31.9%)	271 (33.7%)	153 (52.2%)	<0.01
QRS duration, mean ± SD	97.2 ± 29.1	118.6 ± 17.0	73 ± 7	0.292
LVEDD (mm), mean ± SD	45 ± 5	58 ± 5	53 ± 7	<0.01

*ACEI, angiotensin-converting enzyme inhibitor; AF, atrial fibrillation; AHF, acute heart failure; ARB, angiotensin receptor blocker; ARNI, angiotensin receptor enkephalinase inhibitor; BUN, blood urea nitrogen; CCB, calcium channel blockers; CCI, Charlson comorbidity index; CHD, coronary heart disease; COPD, chronic obstructive pulmonary disease; CysC, Cystatin C; DBP, diastolic blood pressure; eGFR, estimated glomerular filtration rate; External Vad, external validation set; Glu, fasting glucose; HFrEF, heart failure with reduced ejection fraction; Hospital stay, length of hospital stay; Internal Vad, internal validation set; LVEDD, Left ventricular end diastolic diameter; MRA, mineralocorticoid receptor antagonist; NT-proBNP, N-terminal pro brain natriuretic peptide; SBP, systolic blood pressure; RDW-CV, coefficient of variation in red blood cell distribution width; Remoteness, distance of residence from hospital; TC, total cholesterol; UA, uric acid*.

### Lasso and Cox Regression for Model Development

**Figure 3:**
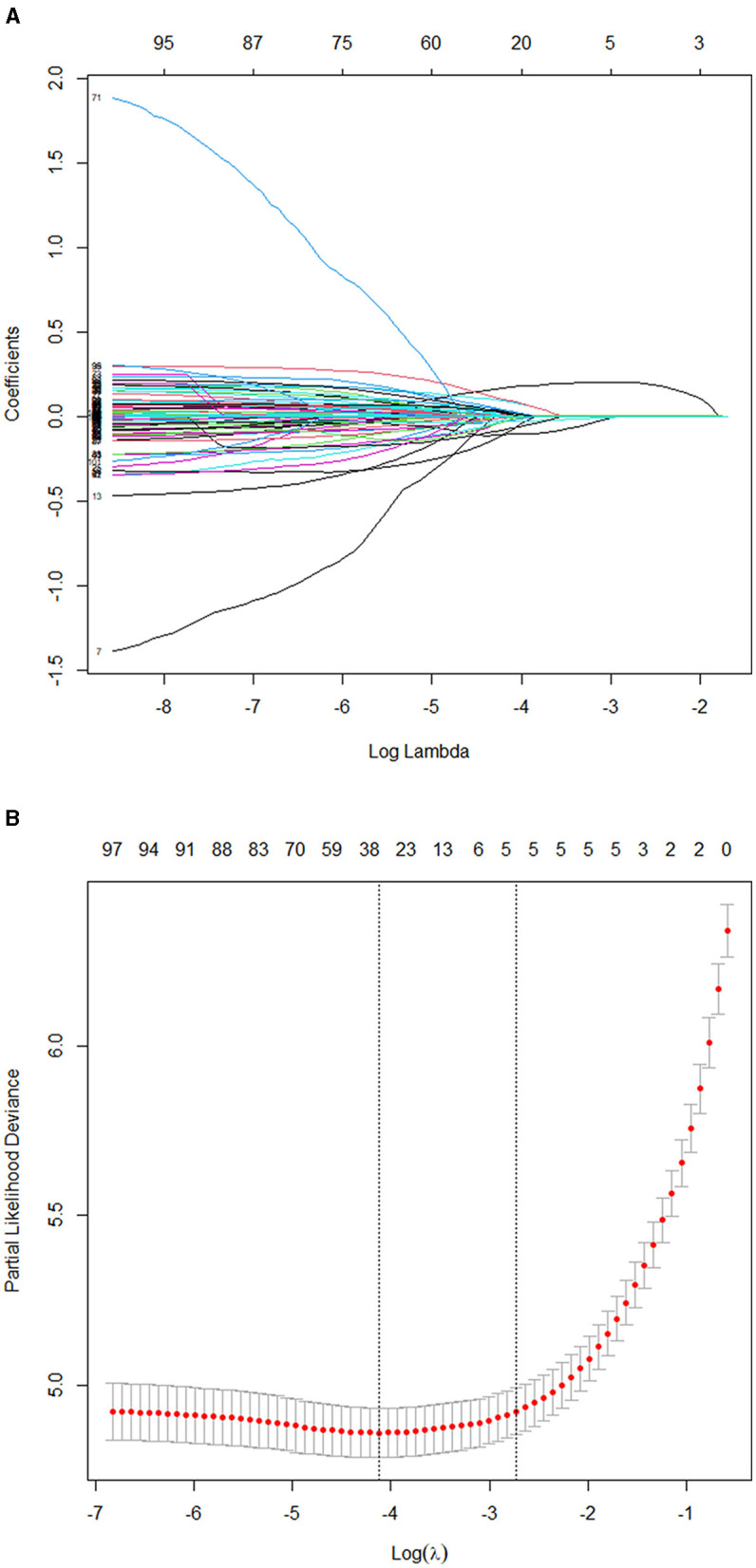
**(A)** Results of the Lasso regression. **(B)** Results of the 10-fold cross-validation. Consequently, five variables were selected for inclusion in the more concise prediction model within one standard error.

The model is developed from 102 variables by screening with the Lasso–Cox regression algorithm. The most prominent advantage of Lasso regression is that it penalizes the coefficients of all variables by regression and makes the relatively unimportant independent variable coefficients zero by the regularization technique. Therefore, when we use Lasso regression, we have retained the punitive and inhibited the collinearity. Results of Lasso regression are shown in Figure 3 [Lasso regression (**A**) and cross-validation (**B**) results].

The five selected variables consisted of AHF, emergency department visit (emergency), eGFR, N-terminal pro-B-type natriuretic peptide (NT-proBNP) and blood urea nitrogen (BUN) level. It supported the proportional risk hypothesis (*p* > 0.05): the *p*-values for AHF, BUN, emergency, eGFR, and NT-proBNP were 0.77, 0.27, 0.38, 0.79, and 0.88, respectively. They were identified as independently associated with the 180-day HF readmission. The results of collinearity diagnosis of the five selected variables are shown in the Table 2. The variance inflation factors are all <10, and the five selected factors preserved very weak multicollinearity.

**Table 2:** Collinearity diagnosis results of five selected variables.

**Variable**	**Tolerance**	**VIF**	***P*-Value**
AHF	0.395	2.532	<0.01^*^
BUN	0.726	1.377	<0.01^*^
Emergency	0.683	1.464	<0.01^*^
eGFR	0.394	2.538	<0.01^*^
NT-proBNP	0.505	1.980	<0.01^*^

*AHF, acute heart failure; BUN, blood urea nitrogen; eGFR, estimated glomerular filtration rate; Emergency, emergency department visit; N-terminal-pro B-type natriuretic peptide; VIF, variance inflation factor*.

* *P < 0.01*.

At the same time, we combined the results with univariate Cox analysis and stepwise regression to avoid missing variables that were clinically significant. According to the results of univariate analysis and stepwise regression, 24 variables were selected from 102 baseline variables, and 24 variables were allocated to five groups (the group was divided into five groups according to the Lasso–Cox result) according to the model inclusion principle of clinical significance (16). In each group, we identified variables with low correlation. They were age (*r* = 0.137), β-blocker usage (*r* = −0.141), hospital discharge way (*r* = 0.136), alcohol taken (*r* = 0.131), and left bundle branch block (*r* = 0.097).

NRI (17) was assessed to evaluate the value of adding the additional clinical variables to the model. NRI showed a significant improvement: 18.5% (95% CI 10.3% to 24.4%) in the development set, 8.2% (95% CI 4.2% to 10.9%) in the internal validation set, and 6.3% (95% CI 2.2% to 7.8%) in the external validation set when adding variables of age and β-blocker. The new model (AHF + emergency + BUN + NT-proBNP + eGFR + age + β-blocker) showed a positive improvement compared with the old model (AHF + emergency + BUN + NT-proBNP + eGFR). However, when adding variables of hospital discharge way, LBBB, and alcohol taken, the model suggested a poor improvement: 2.7 (95% CI 0.5% to 3.6%), 2.6 (95% CI 1.8% to 3.8%), and 0.2 (95% CI −0.2% to 0.5%) (Table 3). It means that some CHF patients cannot be correctly classified.

**Table 3:** Net reclassification improvement in CHF patients who had unplanned readmission in the three patient sets.

**Net reclassification improvement in CHF patients**		
	NRI (%) (AHF +Emergency + BUN + NT-proBNP + eGFR + Age + β-blocker)	NRI (%) (AHF +Emergency + BUN + NT-proBNP + eGFR + Hospital discharge way + LBBB + Alcohol taken)
Training set (AHF + Emergency + BUN + NTproBNP + eGFR)	18.5	2.7
Internal Vad(AHF + Emergency + BUN + NTproBNP + eGFR)	8.2	2.6
External Vad (AHF + Emergency + BUN + NTproBNP + eGFR)	6.3	0.2

*External Vad, external validation set; Internal Vad, internal validation set; NRI, net reclassification improvement*.

Considering the Akaike information criterion (19) and convenience of clinical practice, we finally applied five variables related to HF readmission. The HRs of age, AHF, emergency, β-blocker, and BUN are 1.247 (1.180–1.317, *p* < 0.01), 3.342 (2.632–4.773, *p* < 0.01), 1.201 (1.086–1.329, *p* < 0.01), 0.722 (0.549–0.950, *p* = 0.02), and 1.132 (1.045–1.225, *p* < 0.01), respectively (depicted in Table 4). The C-index in the development set is 0.75 (95% CI: 0.72–0.79; *p* < 0.001).

**Table 4:** Multivariate Cox regression of 180-day unplanned readmission in patients with CHF.

**Variable**	**Hazard ratio (HR)**	**Lower 95%**	**Upper 95%**	***P-*Value**
Age	1.247	1.180	1.317	<0.01^*^
AHF	3.342	2.632	4.773	<0.01^*^
Emergency	1.201	1.086	1.329	<0.01^*^
β-blocker	0.722	0.549	0.950	0.02^*^
BUN	1.132	1.045	1.225	<0.01^*^

*AHF, acute heart failure; β-blocker, beta-blocker usage; BUN, blood urea nitrogen; Emergency, emergency department visit*.

* *P < 0.05*.

**Figure 4:**
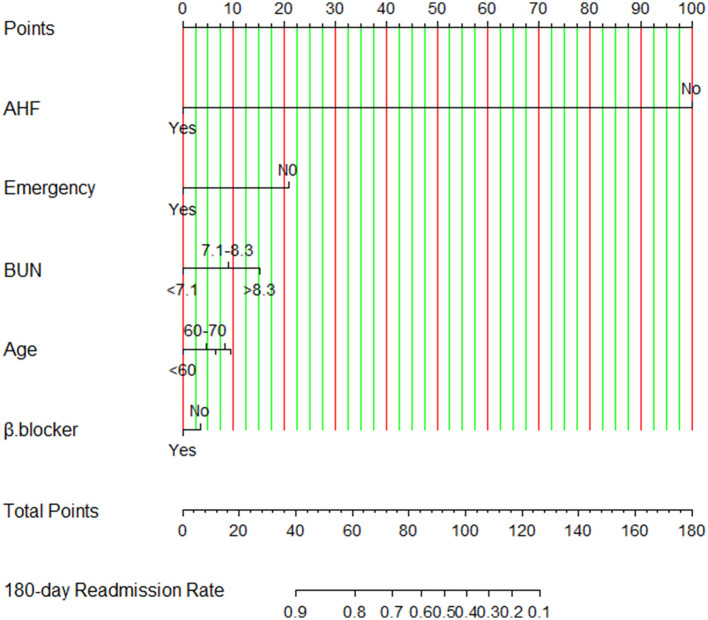
Nomogram for risk of readmission constructed using the selected predictors. Using this nomogram, the individualized 180-day readmission risk for patients with chronic heart failure is easy to calculate. AHF, history of acute heart failure; BUN, blood urea nitrogen level; β-blocker, beta-blocker usage; emergency, emergency department visit.

A nomogram is built to visualize the risk of readmission by using the selected predictors according to the probability values obtained by Cox regression modeling in the development set (Figure 4). Firstly, the values of the predictive factors at baseline were recorded using standard criteria for each patient. Secondly, the score of the patient for each variable is determined by drawing a line perpendicular to the top line. Finally, we add the scores of all selected variables, and the total score is determined by drawing a corresponding line perpendicular to the bottom line to obtain the 180-day HF readmission risk. For example, a patient with a history of emergency admission, with a total of 60 points, has an 80% risk of 180-day readmission according to the nomogram of the study. Meanwhile, we developed an interactively dynamic nomogram online (https://youyueer.shinyapps.io/dynnomapp/).

The validation of the prediction model in the internal validation set is evaluated by discrimination and calibration. A calibration plot further shows good fitness with the actual readmission rates (Supporting Information, Supplementary Figure 1).

### Internal Validation

In order to verify the effectiveness of the nomogram, it is necessary to predict the 180-day readmission of patients with CHF. Besides this, we conducted a comprehensive validation using the internal validation set. The C-index of the internal validation model is 0.75 (95% CI: 0.69–0.81). It should be specially noted that the calibration plot can show good consistency in predicting the 180-day readmission risk for patients with CHF (Supporting Information, Supplementary Figure 1).

### External Validation

We found that AHF, emergency, eGFR, BUN, and NT-proBNP were independently associated with the 180-day readmission of patients with CHF. In order to establish an optimal nomogram model, the C-index is used to validate the model using the external validation set. The C-index is 0.73 (95% CI: 0.64–0.83), indicating that the nomogram could correctly predict individuals with a 180-day unplanned readmission in the external validation set.

## Discussion

For patients with CHF, an accurate prediction of the readmission rate will aid in clinical stratification and treatment decision-making for the development of appropriate diagnosis and treatment programs and the development of healthcare and management guidelines for HF. In this study, we established an easy-to-use nomogram based on easy-to-get clinical variables and validated in patients in a second hospital (>1,000 km away from the first hospital). This ensured wide applicability of the prediction model. This is the first 180-day CHF readmission model based on data of hospitals in North and South China. Compared with previous HF readmission models (8–11), we considered that the lateness of hospitalization in patients with CHF after discharge and the 180-day readmission are more in line with the actual situation.

A 90-day readmission model of HF is developed by Tan et al. from the South China. The C-statistic of the model of Tan is 0.732. The model shows a moderate predictive accuracy; however, 95% CI and validation are not given. Another HF model is developed by Yang et al. and they estimate 30-day (C-index: 0.778, 95% CI: 0.693–0.862) and 1-year readmission rates (C-index: 0.738, 95% CI: 0.640–0.836). The model is developed in North China and lacks external validation. Our nomogram shows a similar accuracy (C-index: 0.752, 95% CI: 0.720–0.790) and we validate it in internal and external sets. However, more mental variables (such as depression, anxiety) and social support should be taken into account in further studies. Han's model (C-index: 0.737, 95% CI: 0.673–0.800) enrolls mental variables, but the common variables of HF and the lack of internal and external validation are neglected. Hughes' analysis of 30- and 180-day readmission showed a lower accuracy (Supplementary Table 1 shows details in the Supplementary Materials).

In terms of validation, our model is validated by cross-validation, internal validation, and external validation. We used 10-fold cross-validation (20), that is, the original samples are divided into 10 groups, each group has several samples, each time a different group is selected for validation, and another nine groups are used to train the model. After the model is trained, the selected group is used to verify the model, and the deviation between the predictive value and the real value of each sample in the group is obtained, and then the average deviation is obtained. According to the above process, the parameter value of the simulation process is the target parameter value when the average deviation is the smallest for 10 cycles. At the same time, the sample is split 7:3 to complete the internal verification. Finally, the validity and consistency of the model are verified in the external set.

In terms of applicability, we used two CHF prospective study cohorts from HF centers in both North and South China, an optimal prognostic prediction model for 180-day readmission, including age, AHF, β-blocker, BUN, and emergency. Different data sources ensure the general applicability of the model. Besides, Lasso regression made the coefficients of some useless variable features penalized and even made some coefficients with smaller absolute values directly forced to zero. Finally, the penalty condition was satisfied and the sum of residual squares was minimized so as to enhance the generalization ability of the model.

Lasso regression used the following formula (21):

$${\hat{\beta }}^{Lasso}=\sum_{i=1}^{n}(y_{i}-\sum_{j=1}^{p}x_{ij}\beta_{j}{)}^{2}+\lambda \sum_{j=1}^{p}\left|\beta_{j}\right|$$

Penalty condition: $\overset{p}{\underset{j=1}{\sum }}|\beta_{j}|\leq \lambda$; the penalty function is to punish the absolute value of the regression coefficient, which requires that the sum of the absolute values of all regression coefficients is less than or equal to the penalty coefficient lambda; *p* refers to the number of the variable; *n* refers to the number of samples. Satisfactory consistency with good calibration was observed in the independent external validation set. The developed nomogram robustly quantified the risk of readmission of an individual within 180 days of discharge. The intuitive features easily allowed the clinical staffs to predict the readmission and prognosis of patients with CHF using several important symptoms and clinical indicators.

### History of CHF

Prediction variables independently associated with readmission are included in our model. Two predictors (AHF and emergency) are associated with the medical history of the patients. AHF (22) is common in the natural history of HF, and it is associated with high in-hospital mortality (23). The occurrence of AHF will lead patients to enter a state of stress. The state of stress will aggravate AHF and atrial fibrillation (24). AHF attack is caused by amplified leukocytes/neutrophils and monocytes/macrophages (25). Neutrophilic leukocytosis (neutrophilia) and continuous activation of neutrophils are the main factors determining the overactive inflammation of AHF and the prognosis of long-term CHF. This keeps patients with CHF in a state of persistent inflammation. A history of AHF suggests that it usually occurred within the past 3 months. Generally speaking, early AHF is an independent risk factor for acute exacerbation in patients with CHF and this is consistent with a previous study of AHF (26), which suggests that the prognosis of patients with CHF is poor at some degree. A Denmark nationwide study showed that patients with acute attack of CHF have higher all-cause mortality and readmission rates than patients with new-onset AHF (27). This may because CHF patients with AHF attack often have more comorbidities. However, AHF recurrence may be reduced if given early and timely attention, and technical clinicians are able to provide too much comprehensive and effective treatment measures.

The emergency treatments are important indicators in the 180-day readmission model for CHF. The emergency department is the key point of initial treatment for patients with AHF attack. The emergency department plays an important role in the management and treatment of AHF patients (28). Therefore, whether CHF patients have AHF attacks and whether they have been to the emergency department are closely related to readmission, as we found in our studies (22).

### Age and NT-proBNP

With the aging process, the number of CHF patients is increasing. In developed countries, the incidence rate of HF is about 1–2% in adults, while in elderly people over 70, the figure rises to nearly 10% (29). The prevalence of HF over 35 years old is 1.3% in China (7), and considering the population base, there are about 13.7 million patients with HF. In addition to aging combined with various diseases, we cannot ignore the problem of frailty. The FRAIL-HF study has shown that patients with frailty had a higher risk of 30-day dysfunction and0 higher 1-year readmission rate and 1-year all-cause mortality (30). This is one of the reasons for CHF readmission. In addition, the elderly have higher levels of plasma natriuretic peptide. NT-proBNP varies with age and cardiac dysfunction. It follows from previous studies that plasma NT-proBNP level is an important biomarker in predicting the prognosis of patients with CHF, especially for patients with diabetes (31), where NT-proBNP is closely related to the left ventricular reconstruction in CHF. However, the NT-proBNP level varies greatly after finishing the HF therapy, accompanied with apparent individualization. In our study, we regard the elevated NT-proBNP level as a baseline predictor, which seems not appropriate at some degree. We should regard the NT-proBNP level as an independent, dynamic biomarker. Only in this way can we get a more accurate readmission rate. ΔNT-proBNP or the ratio of BNP/NT-proBNP could be observed and calculated in the future prediction model.

### Renal Dysfunction and CHF

In the present study, we mainly consider plasma BUN level. The level of BUN reflects the protein metabolism, indicating the renal function, which is independently associated with the prognosis of patients with CHF. Moreover, they are also selected for model inclusion. Fluid overload is a common pathophysiological mechanism of CHF as well as renal disorders. There exist complex neurohormonal interactions between the heart and kidney for patients with CHF. Patients with CHF have a higher incidence of renal dysfunction due to several shared pathophysiological pathways and mutual risk factors (32). The existing results suggest that renal dysfunction progression is closely related to readmission rate and clinical prognosis. For patients with CHF, the hemodynamic perturbations would lead to sodium and water retention and worsening of renal function. In addition, the cardiorenal syndrome further highlights the concept of bidirectional interaction, such that CHF has several negative effects on kidney function, while renal dysfunction significantly influences cardiac function. As the course of CHF progresses, blood flow will decrease through tissues and organs. It should be noted that the kidney is sensitive to ischemia, resulting in significant decreases in eGFR, as well as glomerular and secondary tubular injuries, activation of the neuroendocrine system (renin–angiotensin–aldosterone system and sympathetic nervous system), congestion of the venous system, and increased central venous pressure. In recent years, studies have shown that an increase of central venous pressure is associated with a poor prognosis for patients with CHF, potentially because a disproportionately higher central venous pressure effectively contributes to elevated left-sided filling pressure (14).

In order to design new strategies to improve clinical outcomes, a better understanding of the mechanisms has to be achieved, and the experimental results for the hemodynamic changes affecting the lungs and kidneys in HF need to be validated further.

eGFR is another factor to be analyzed; however, it is a sensitive factor together with the change of blood creatinine. Improvement is not obvious when we add eGFR to our prediction model. This is similar to the conclusion of an HFpEF study (26). This may because of the fluctuation of eGFR, which leads to inaccurate results. Compared with this study, our present study included a small number of ultrasound variables and future research should be evaluated in detail.

### Usage of β-Blocker and CHF

From the analysis, we observed the benefits of β-blocker usage in CHF patients. In future studies, we would consider the dosage of β-blockers in detail, as well as the individual differences when choosing the requirements of drug use. The elderly aged ≥75 years who received a β-blocker hold a lower 90-day mortality rate and lower readmission rate (33). Consequently, β-blocker therapy is closely related to the prognosis of patients with HF.

Finally, the five variables associated with CHF readmission entered into the model were as follows: AHF, emergency, age, BUN, and β-blocker usage. This fits with the AIC principle. When the predictive effect is similar, we tend to choose fewer variables for clinical applicability.

## Study Strengths and Limitations

Several studies focused on the enhancement of the accuracy of the models (34) but ignored their wide clinical applications. Our study was conducted in a real-world situation. By conducting a rigorous multicenter prospective study, this work focuses on developing a prediction model for a 180-day CHF readmission using standard and easily collected clinical variables. The prediction model can be well-validated in an external database from a second HF center. As a result, the proposed predictor model will play a key role for effective verification. Nevertheless, this study has several potential limitations. Firstly, we did not take into account the all-cause mortality endpoint completely. Because of the influence of COVID-19, accurate mortality might not have been observed at some degree. In a future study, we will take into account heart injury as a result of the new coronavirus (35–37). Secondly, the model does not consider some novel biomarker phenotypes associated with CHF. We are considering whether adding some novel biomarker phenotypes can increase the accuracy of model prediction.

## Conclusions

This work mainly provides a simple model, which can address data collected for clinical practice to predict the 180-day readmission of patients with CHF. Both internal and external validations further suggest a broad applicability of the given model. From the results, the prediction model can show a reference value for a meaningful prognosis to stratify patients with CHF and then aid in timely clinical decision-making.

## Data Availability Statement

The raw data supporting the conclusions of this article will be made available by the authors, without undue reservation.

## Ethics Statement

The studies involving human participants were reviewed and approved by Qingdao Central Hospital Ethics Committee, The Ethics Committee of the First Affiliated Hospital of Shantou University Medical College. The patients/participants provided their written informed consent to participate in this study.

## Author Contributions

SG: conception and methodology, project administration, and writing of the original draft. GY: conception and methodology and data curation (from the North China Heart Failure Center). QX: conception and methodology and data curation (from the North China Heart Failure Center). GW: conception and methodology and data curation (from the South China Heart Failure Center). XT: conception and methodology and data curation (from the South China Heart Failure Center). JZ: writing, review, and editing. NL: writing, review, and editing. All authors contributed to the article and approved the submitted version.

## Funding

This study was supported by the Guangdong High-Level University Development Program Research Fund (2020) and Innovation Team Project of Guangdong Universities, China (Natural, No. 2019KCXTD003).

## Conflict of Interest

The authors declare that the research was conducted in the absence of any commercial or financial relationships that could be construed as a potential conflict of interest.

## Publisher's Note

All claims expressed in this article are solely those of the authors and do not necessarily represent those of their affiliated organizations, or those of the publisher, the editors and the reviewers. Any product that may be evaluated in this article, or claim that may be made by its manufacturer, is not guaranteed or endorsed by the publisher.
