# Supplementary material for: Development and Validation of a Nomogram to Predict the 180-Day Readmission Risk for Chronic Heart Failure: A Multicenter Prospective Study
Source: Front Cardiovasc Med. 2021 Sep 7;8:731730. doi: 10.3389/fcvm.2021.731730 (PMC8452908; doi:10.3389/fcvm.2021.731730)
Supplement: Supplementary file 1 [file Data_Sheet_1.doc]

**Supplementary Material for ‘Development and validation of a nomogram to predict the 180-day readmission risk for chronic heart failure: a multi-center prospective study’**

**Authors:** Shanshan Gao, Gang Yin, Qing Xia, Guihai Wu, Jinxiu Zhu, Nan Lu, Jingyi Yan, Xuerui Tan

**Figure S1.** Calibration plots in the development and validation sets.

1. Calibration plot for the development set,


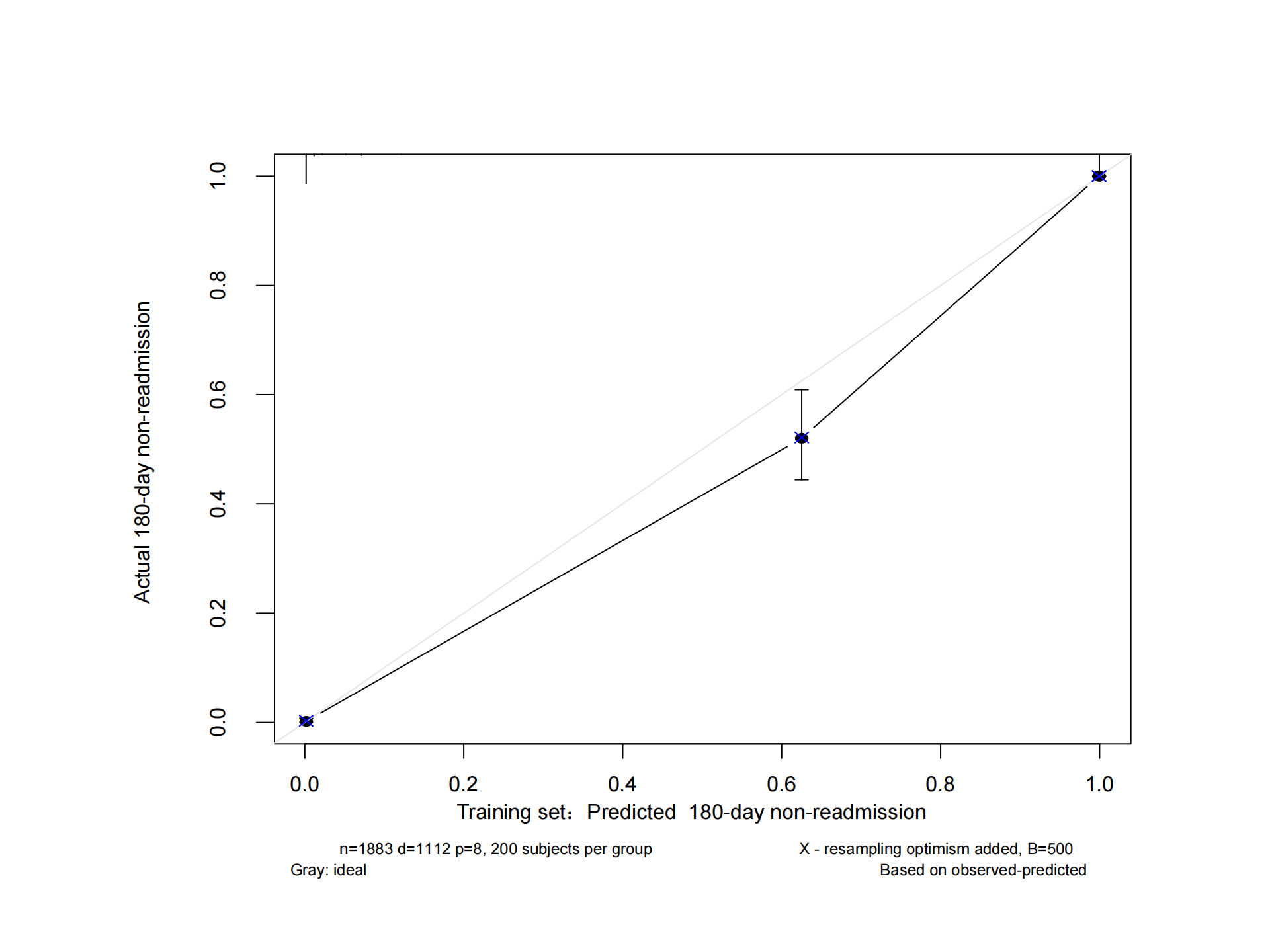


1. Calibration plot for the internal validation set,


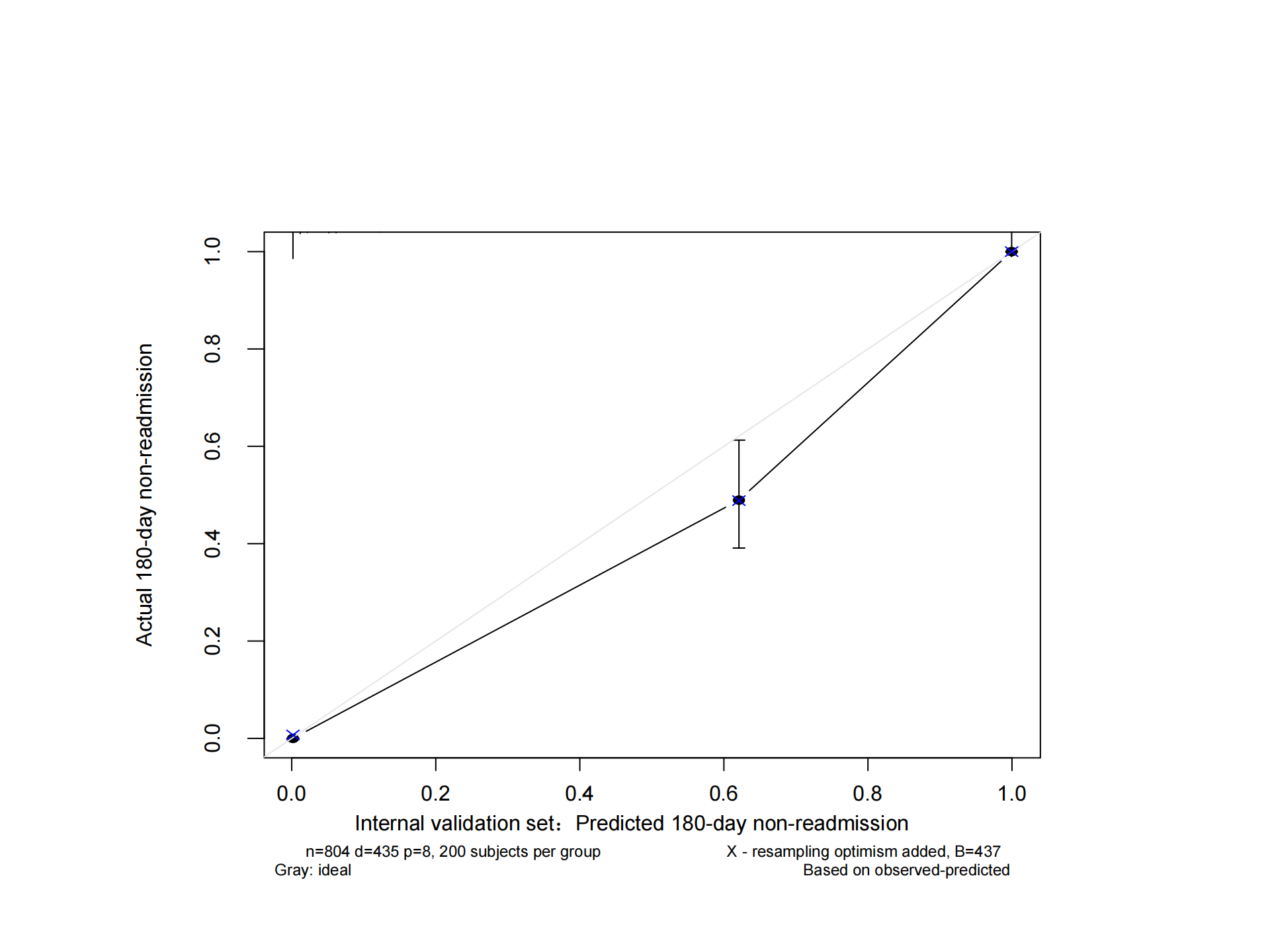


1. Calibration plot for the external validation set.


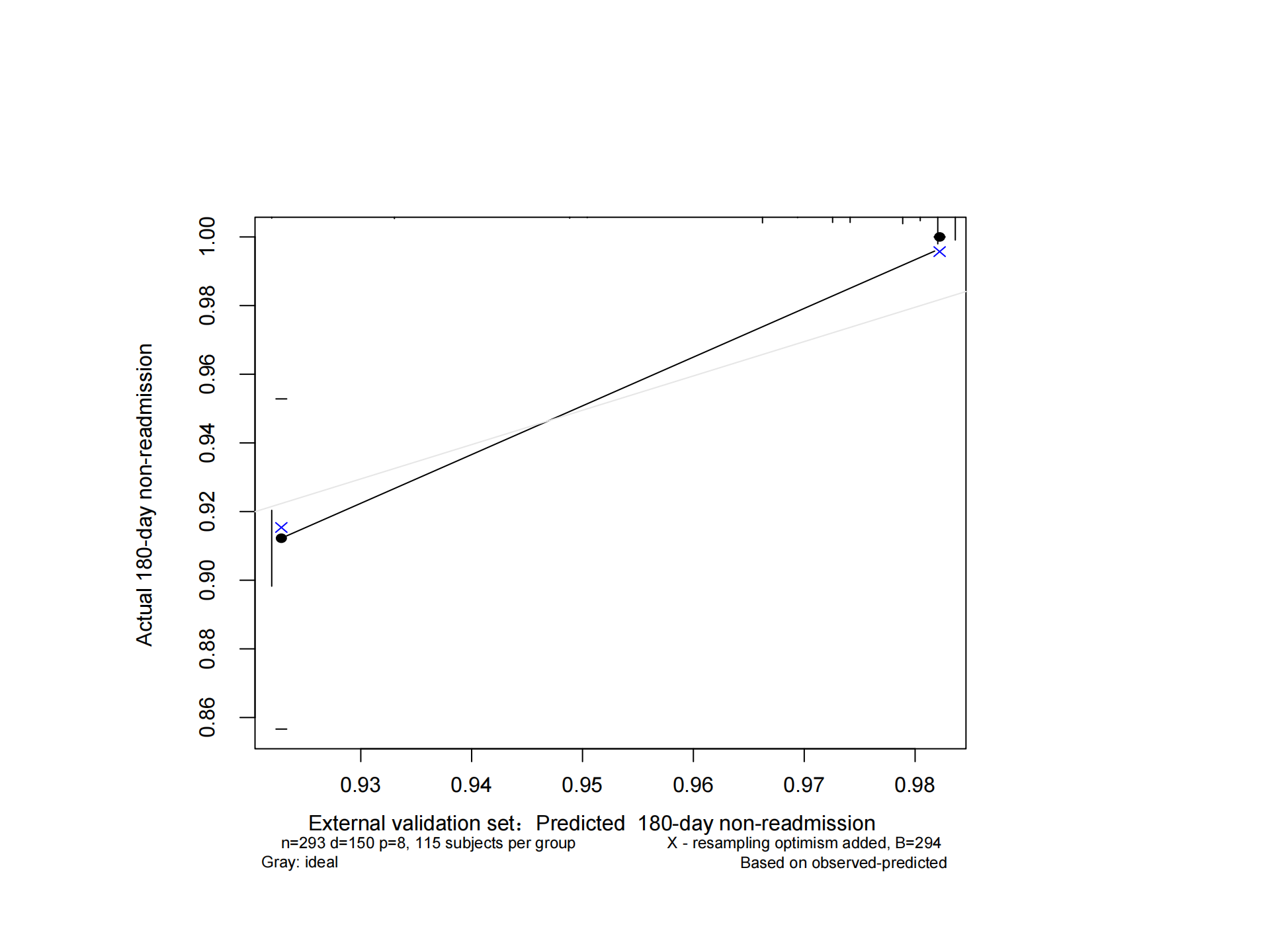


**Table S1. The comparisons of C-index among models**

|  | development |  | internal |  | external |  |
| --- | --- | --- | --- | --- | --- | --- |
| Models | C-index | 95%CI | C-index | 95%CI | C-index | 95%CI |
| Our nomogram | 0.752 | 0.720-0.790 | 0.750  (bootstrap in internal set) | 0.690-0.810 | 0.731  (bootstrap in external set) | 0.640-0.830 |
| Tan (90-day readmission) | 0.732 | _ | _ | _ | _ | _ |
| Yang (30-day readmission, 1-year readmission) | 0.778,  0.738 | 0.693-0.862,  0.640-0.836 | 0.778 ,  0.738  (bootstrap) | 0.693-0.862,  0.640-0.836 | _ | _ |
| Han | 0.737 | 0.673-0.800 | _ | _ | _ | _ |
| Hughes (30-day readmission, 180-day readmission) | 0.640,  0.590 | 0.600-0.680,  0.570-0.610 | _ | _ | _ | _ |
